# Simultaneous embolization of the right portal and hepatic veins before intrahepatic cholangiocarcinoma resection

**DOI:** 10.31744/einstein_journal/2024RC0524

**Published:** 2024-10-17

**Authors:** Américo Gusmão Amorim, Olival Cirilo Lucena da Fonseca, Raimundo Hugo Matias Furtado, Laécio Leitão Batista, Ludmilla Rodrigues Oliveira Costa, Igor Montenegro Galvão

**Affiliations:** 1 Universidade de Pernambuco Hospital Universitário Oswaldo Cruz Recife PE Brazil Hospital Universitário Oswaldo Cruz, Universidade de Pernambuco, Recife, PE, Brazil.; 2 Universidade de Pernambuco Faculdade de Ciências Médicas de Pernambuco Recife PE Brazil Faculdade de Ciências Médicas de Pernambuco, Universidade de Pernambuco, Recife, PE, Brazil.

**Keywords:** Cholangiocarcinoma, Hepatectomy, Bile duct neoplasms, Embolization, therapeutic, Hypertrophy

## Abstract

Major liver resections require extensive margins. Occasionally, insufficient parenchyma is available after surgery to maintain liver function. In such cases, vascular embolization in the affected lobe is necessary to induce contralateral lobe hypertrophy. We present a case of embolization of the right portal and hepatic veins prior to intrahepatic cholangiocarcinoma resection. Embolization was performed because of insufficient residual parenchyma on imaging studies. The patient recovered well with no signs of liver failure, and remains in remission at 3 years postoperatively. Knowledge of the use of this technique in association with surgical resection can reduce postoperative complications and allow the removal of larger tumors than those previously considered borderline.

## INTRODUCTION

Cholangiocarcinoma is a rare adenocarcinoma of the biliary duct, classified as perihilar, intrahepatic, or extrahepatic. The tumor typically progresses rapidly and has nonspecific clinical findings, which postpones diagnosis.^(1)^

Surgical resection of the mass is the only curative approach; however, few patients are eligible for treatment. The presence of free margins with sufficient remaining hepatic parenchyma to maintain liver function is a challenge in this procedure.^(2)^ Among the new surgical strategies, embolization of the portal and hepatic veins has become a popular alternative for treating hypertrophy of the contralateral hepatic lobe. Although this technique has yielded satisfactory results, there is no consensus regarding its use.^(3)^

The present case of intrahepatic cholangiocarcinoma in a patient of an unusual age for this type of tumor was treated via simultaneous embolization of the hepatic and portal veins with posterior surgical resection.

## CASE REPORT

The patient was a 41-year-old female who underwent total abdominal ultrasound, which showed a solid hypoechoic nodular image in the right hepatic lobule, with regular contours, measuring 3.2 × 2.5cm; the liver was of normal dimensions with homogeneous parenchymal echotexture and normal intrahepatic biliary ducts. The patient denied any abdominal complaints, weight loss, comorbidities, previous surgeries, or a family history of neoplasms. Informed consent was obtained from the patient for the publication of this report.

Subsequently, magnetic resonance imaging (MRI) of the upper abdomen was performed, which revealed a lobulated hepatic nodule, measuring 3.1 × 3.5cm between lobes VII and VIII. The contrast-enhanced ultrasound liver reporting and data system (CEUS LI-RADS) indicated that the mass was probably or definitely malignant and not HCC-specific (LR-M category).

Liver biopsy and immunohistochemical studies revealed hepatic tissue partially substituted for immature neoplasm, with a probable origin in the biliopancreatic tract. The main diagnostic hypothesis was cholangiocarcinoma.

Therefore, the surgical and radiological team opted for a lobe hepatectomy associated with simultaneous embolization of the right portal and hepatic veins as a strategy to induce liver hypertrophy and gain sufficient parenchyma to safely resect the involved lobe and reduce the risk of hepatic failure and insufficient remaining tissue for vital functions.

Considering the aggressive characteristics of the tumor and the good prognosis, simultaneous embolization of the portal and hepatic vessels was performed. Contrast-enhanced right and middle hepatic phlebography was performed via jugular access and guided by Doppler ultrasound. A 22 × 18mm vascular plug was released into the right hepatic vein. Subsequently, the portal branch of segment VI was punctured peripherally via right lateral abdominal access and guided by hepatic Doppler ultrasound, thus performing direct portography with non-ionic contrast. After insufflation of the 8 × 40mm balloon for backflow protection, glue was injected into a microcatheter to occlude the right portal segments of the right lobe.

**Figure 1:**
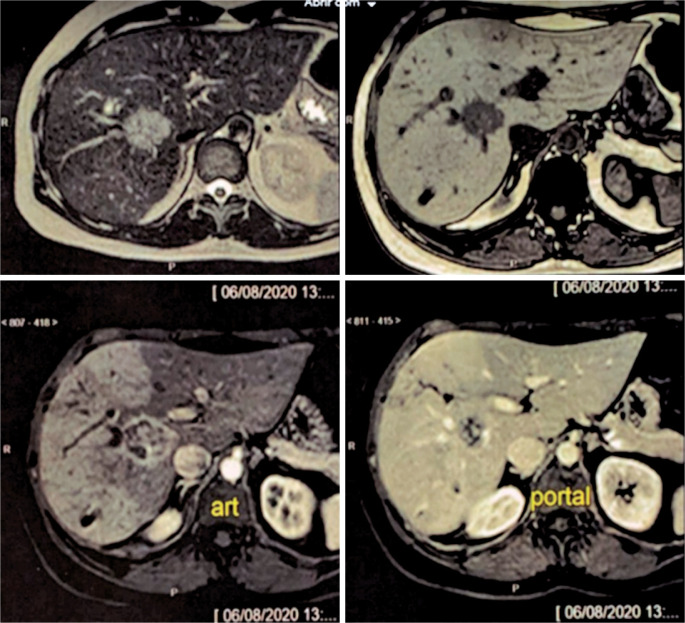
Magnetic resonance imaging after embolization showing occlusion of the right hepatic vein and the right branch of the portal vein

**Figure 2:**
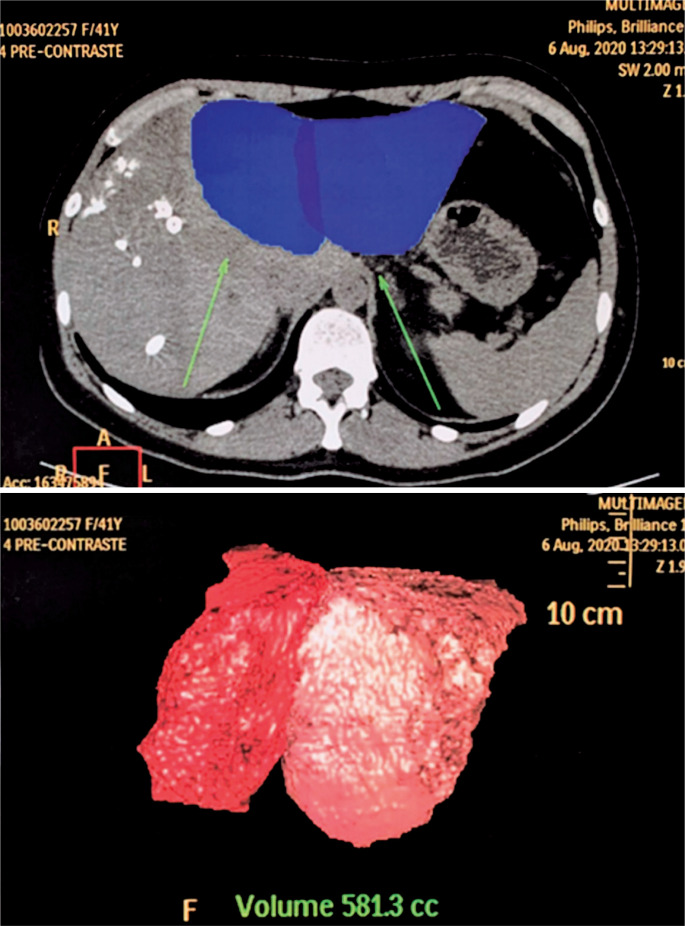
Volumetry of the remaining liver (left lobe) after right hepatic and portal vein embolization (axial slice and 3D reconstruction)

Occlusion of the right hepatic vein and the right branch of the portal vein was noted on MRI after embolization, with the accessory segmental vein persisting and draining segments V and VIII (Figure 1). The left branch of the portal vein and hepatic veins of the left lobe showed normal flow. Volumetry of the left lobe, performed 21 days after the procedure, showed segments II and III as measuring 346cm^3^ and segment IV as measuring 250cm^3^, totaling 581cm^3^ (Figure 2).

Finally, 42 days after simultaneous embolization, a right hepatectomy was performed with ligation of the right branch of the portal vein and the right hepatic vein. All surgical margins were preserved ≥0.1cm away from the tumor. The patient recovered well with no signs of liver failure.

**Figure 3:**
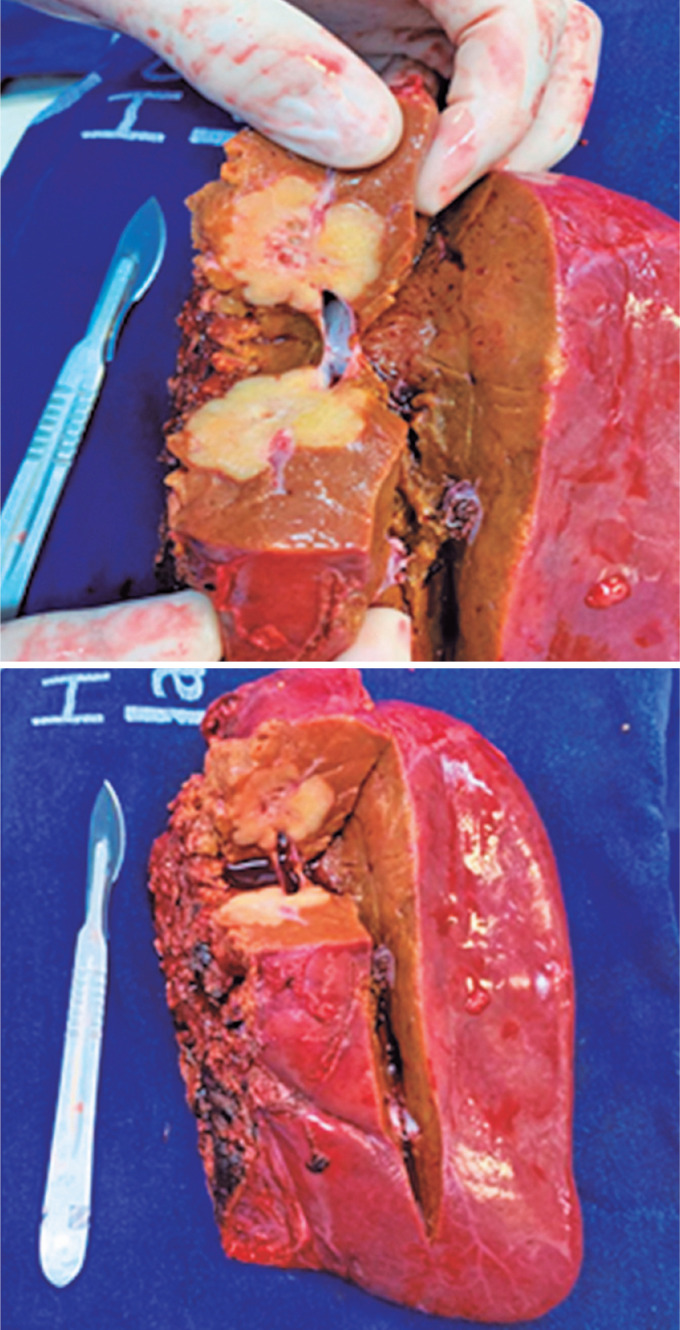
(A and B) Right liver lobe with an intrahepatic nodular mass around the right portal vein branch, after resection

The surgical specimen weighed 663.27g and measured 17 × 12 × 8.5cm (Figure 3A and B). Histopathological examination revealed a unifocal tumor of 3 × 3 × 2.8cm, without vascular or perineural invasion, with dilatation of the bile ducts adjacent to the tumor. The diagnosis of the biopsy of the right lobe was a well-differentiated, grade 1, unifocal intrahepatic cholangiocarcinoma, which was restricted to the liver, with the lymph node free of neoplasia. The final TNM staging was pT1aN0Mx. An estimated 41.7% increase in left lobe hypertrophy and parenchymal gain was noted. The patient has maintained a cancer-free status in remains in remission. Following surgical resection, she underwent five cycles of capecitabine as adjuvant therapy and continues to attend regular follow-up appointments at 3 years postoperatively, ensuring sustained cancer-free survival.

The study was approved by the Research Ethics Committee of the *Complexo Hospitalar Hospital Universitário Oswaldo Cruz* (HUOC)/ *Pronto-Socorro Cardiológico Universitário de Pernambuco - Prof. Luiz Tavares* (PROCAPE) CAAE: 68457323.8.0000.5192; 5,982,713.

## DISCUSSION

Embolization of the portal vein before major liver resection (perihilar tumors, intrahepatic cholangiocarcinomas, and hepatocarcinomas) is a common approach to induce hypertrophy of the residual liver and preserve postoperative function. However, the required remaining liver volume is typically not achieved within the expected 2 week time period, thereby necessitating subsequent embolization of the hepatic vein.^(1,4)^ Studies have demonstrated that hepatic vein embolization following portal vein embolization can increase hypertrophy of the target hepatic lobe by up to 44.2% of the original volume, compared to only 24.8% for portal vein embolization alone.^(5)^

A limitation of sequential embolization is the delay in performing the hepatectomy, which requires two preoperative steps. One study revealed that 25.3% of patients who underwent sequential embolization were ineligible for resection, which is one of the most common reasons for tumor progression.^(2,3)^

To improve efficiency and facilitate surgical resection, modifications of the technique suggest simultaneous portal and hepatic vein embolization. Despite occlusion of the venous branches for irrigation and drainage in the target lobule, the distal branches can be irrigated through arteriovenous shunts, which would cause atrophy of the target segment without necrosis.^(4)^ Thus, the technique is possible without causing deterioration of liver function.^(3)^ Data indicate a significant gain of 61.2% in the remnant volume compared to only 28.9% following portal vein embolization.^(6)^ In our case, liver hypertrophy was 41.7% of the original size, which was sufficient for the patient to thrive after surgery without any adverse events.

Occasionally, complications secondary to embolization are reported, such as embolization of the wrong target trunk of the hepatic vein without major negative repercussions. The most common causes of technique failure are insufficient hypertrophy of the remaining lobe or rapid tumor progression without the possibility of surgical resection.^(3)^ One peculiar characteristic of intrahepatic cholangiocarcinoma embolization is the possible induction of tumor mass enlargement via the secretion of growth factors from the hypertrophied lobe, which was not observed in our case.^(7)^

Intrahepatic cholangiocarcinoma is incidentally diagnosed in 20-25% of cases via complementary imaging examinations.^(8)^ In our case, the patient was diagnosed via routine abdominal ultrasound at an unusual age (41 years), with this tumor being most prevalent in those aged ≥65 years.^(9)^

## CONCLUSION

The dimensions of the nodule indicated that hepatectomy alone would not be sufficient to safeguard the liver parenchyma. Therefore, simultaneous embolization of the portal and hepatic veins was essential for good surgical outcomes in this case.
